# *Legionella* colonization and 3D spatial location within a *Pseudomonas* biofilm

**DOI:** 10.1038/s41598-024-67712-4

**Published:** 2024-07-22

**Authors:** Ana Rosa Silva, Luis F. Melo, C. William Keevil, Ana Pereira

**Affiliations:** 1 LEPABE - Laboratory for Process Engineering, Environment, Biotechnology and Energy, Faculty of Engineering, University of Porto, Rua Dr. Roberto Frias, 4200-465 Porto, Portugal; 2 ALiCE - Associate Laboratory in Chemical Engineering, Faculty of Engineering, University of Porto, Rua Dr. Roberto Frias, 4200-465 Porto, Portugal; 3 School of Biological Sciences, University of Southampton, Southampton, UK

**Keywords:** Biofilm, Biofilm structure, *Legionella pneumophila*, *Legionella* spatial location, Stagnation, Water systems, Microbiology, Environmental sciences

## Abstract

Biofilms are known to be critical for *Legionella* settlement in engineered water systems and are often associated with Legionnaire’s Disease events. One of the key features of biofilms is their heterogeneous three-dimensional structure which supports the establishment of microbial interactions and confers protection to microorganisms. This work addresses the impact of *Legionella pneumophila* colonization of a *Pseudomonas fluorescens* biofilm, as information about the interactions between *Legionella* and biofilm structures is scarce. It combines a set of meso- and microscale biofilm analyses (Optical Coherence Tomography, Episcopic Differential Interference Contrast coupled with Epifluorescence Microscopy and Confocal Laser Scanning Microscopy) with PNA-FISH labelled *L. pneumophila* to tackle the following questions: (a) does the biofilm structure change upon *L. pneumophila* biofilm colonization?; (b) what happens to *L. pneumophila* within the biofilm over time and (c) where is *L. pneumophila* preferentially located within the biofilm? Results showed that *P. fluorescens* structure did not significantly change upon *L. pneumophila* colonization, indicating the competitive advantage of the first colonizer. Imaging of PNA-labelled *L. pneumophila* showed that compared to standard culture recovery it colonized to a greater extent the 3-day-old *P. fluorescens* biofilms, presumably entering in VBNC state by the end of the experiment. *L. pneumophila* was mostly located in the bottom regions of the biofilm, which is consistent with the physiological requirements of both bacteria and confers enhanced *Legionella* protection against external aggressions. The present study provides an expedited methodological approach to address specific systematic laboratory studies concerning the interactions between *L. pneumophila* and biofilm structure that can provide, in the future, insights for public health *Legionella* management of water systems.

## Introduction

*Legionella pneumophila* is a well-known waterborne pathogen responsible for the severe, and often fatal, pneumonia named Legionnaires’ Disease^1,2^. *L. pneumophila* is a very intriguing and complex microorganism which exhibits multiple adaptation and survival mechanisms in the environment, according to the conditions to which it is exposed^2–4^.

Protozoa and biofilms are reported as key ecological niches for *Legionella* settlement and survival in water systems^5^. Protozoa are known to graze the microcolonies of the biofilm, in a prey-predator relationship, and are able to shape the microbial community including the number of pathogens^1,6^. However, the specific role of biofilms in *Legionella* survival and replication in biofilms is not consensually accepted among researchers^2,4,6^. While some researchers advocate that *Legionella* growth requires a protozoan host^7,8^, others argue that *Legionella* is able to colonize and survive in biofilms without intracellular replication^9,10^. Rogers et al.^11^ and Wadowsky et al.^12^ stated that the presence of non-legionellae bacteria could favor *Legionella* growth. Later, Surman et al.^9^ while using a model water system showed that *L. pneumophila* was able to proliferate within biofilms without protozoan intracellular replication, as long as other bacterial species were present. More recently, Stewart et al.^13^ showed that biofilms composed of *Klebsiella pneumoniae* and *Flavobacterium* sp. allowed *Legionella* persistence for long periods.

Biofilms are complex three-dimensional (3D) heterogeneous structures of microorganisms encased in self-produced extracellular polymeric substances (EPS)^5,14^. Engineered water systems are complex networks that offer multiple localized conditions, including different temperatures, surface materials, hydrodynamics and nutrients that can favour biofilm formation^15,16^. Such conditions are known to affect the characteristics of the biofilms, including its microbiome^17–19^, its structure^20^, and how *Legionella* colonizes such biofilms^21–23^. For example, biofilms grown under stagnation are usually thicker, less compact, and more susceptible to sloughing-off^20,24^. Besides, water stagnation has also been reported to be critical for *Legionella* proliferation, due to repeated failures in disinfection procedures and higher accumulation of nutrients^23^.

From a public health perspective, it is important to investigate *Legionella* colonization and the spatial location within an existing biofilm structure. The risk for legionellosis will be different if *Legionella* is located on the outer regions of the biofilm, where it is more susceptible to slough-off and release into the bulk water, or if positioned closer to the bottom regions of the biofilm, where *Legionella* is expected to be more protected against disinfection procedures. Biofilm slough-off can release significant amounts of *Legionella* into the bulk water, which through aerosolization settings (like cooling towers or showers, etc.) can reach human lungs and trigger Legionnaire’s Disease^5^. Very little information is available on the role of biofilm structure on *Legionella* colonization. For example, Shen et al.^25^ investigated the relationship between biofilm structure and *Legionella* adhesion and detachment from biofilms. The authors reported that biofilm roughness was found to favor *L. pneumophila* adhesion to the biofilm top surface. However, most investigations have been focused on evaluating the effect of plumbing materials, temperature and microbial consortia on biofilm colonization by legionellae^11,26,27^.

The present work uses an expedited, high-throughput and reproducible model, comprising a 12-well plate platform, a monospecies *Pseudomonas fluorescens* biofilm, in combination with molecular tracking with a specific 16S rRNA peptide nucleic acid (PNA) probe for *L. pneumophila* detection^28^, and 3D imaging techniques (Optical Coherence Tomography—OCT, Episcopic Differential Interference Contrast with Epifluorescence—EDIC/EF—microscopy, and Confocal Laser Scanning Microscopy—CLSM). The model does not mimic biofilms, *Legionella* behaviour, nor *Legionella*-biofilms interactions in real-field engineered water systems. Rather, the model uses a bacterium commonly found in biofilms of engineered water systems^13,21^ and is well characterized regarding biofilm formation^20,24,29^. It also considers the conditions that are known to favour *Pseudomonas fluorescens* biofilm build-up like temperature (30 °C) and formulated low nutrient medium (R2), that are not optimum for *Legionella* growth^30^. This methodological approach aims to understand how *L. pneumophila* colonization of *P. fluorescens* biofilms affects the overall biofilm structure as well as the spatial location of *Legionella* within the biofilm.

## Materials and methods

### Bacterial strains and culture maintenance

The bacterium used to form the biofilms was *P. fluorescens* ATCC 13525^T^. Bacteria were grown overnight at 30 ± 3 °C under agitation in 100 mL of sterile R2 (0.5 g/L peptone, 0.5 g/L glucose, 0.1 g/L magnesium sulphate · 7H_2_O, 0.3 g/L sodium pyruvate, 0.5 g/L yeast extract, 0.5 g/L casein hydrolysate, 0.5 g/L starch soluble and 0.393 g/L di-potassium phosphate·3H_2_O). All components were purchased from Merck (Darmstadt, Germany).

*L. pneumophila* serogroup 1 (WDCM00107), an environmental isolate, was used throughout this work. The choice relied on the fact that *L. pneumophila* is responsible for approximately 90% of the reported cases of legionellosis^21^. Bacteria was grown on buffered charcoal-yeast extract (BCYE) agar (Merck, Portugal) at 37 °C for 2 days.

### Preparation of the biofilm set-up

In this study, polyvinyl chloride (PVC) coupons placed inside 12-well plates were used to grow biofilms. PVC was selected since it is often found in water engineered systems and past studies showed that it supports biofilms colonized by *Legionella*^11^. Coupons were sonicated in a 10% sodium dodecyl sulphate (VWR International, Portugal) solution for 5 min. To remove any remaining detergent, coupons were rinsed with tap water and then sonicated again in ultrapure water. Afterwards, the surfaces were rinsed in ultrapure water, air dried, and sterilized with ultraviolet (UV) radiation (254 nm) for 60 min each side. Double-sided adhesive tape was placed in each plate well, sterilized with UV radiation for 60 min, and finally, the sterile coupons were glued in place.

### Biofilm formation and *Legionella* spiking

An overnight culture of *P. fluorescens* ATCC 13525^T^ was harvested by centrifugation at 4000 rpm for 10 min at 25 °C (MegaStar 600R, VWR International, Portugal). Cell concentration was adjusted to an optical density (OD_610_ nm) of 0.7 in fresh R2, which is equivalent to approximately 10^8^ colony-forming units per mL (CFU/mL).

Each well was filled with 3 mL of the prepared bacterial suspension. The plates were then incubated for 14 days at 30 °C under stagnation. Three days after starting biofilm formation, biofilms were spiked with a suspension of *L. pneumophila* containing 10^9^ CFU/mL and incubated again under the same conditions. Culture media was replaced by fresh R2 every 2 days.

### Biofilm sampling

Coupons were sampled after 3, 4, 7, 9, 11 and 14 days for biofilm analysis. In the 12-well plates, the bulk media was gently removed and rinsed with sterile saline solution (8.5 g/L) to remove planktonic cells. Coupons were kept in saline solution or let to air dry for imaging (detailed procedures described in "Optical coherence tomography (OCT)" and "Peptide nucleic acid (PNA) – Fluorescence in situ hybridization (FISH)" sections). For quantification of the sessile cells in the biofilms, coupons were gently removed from the 12-well plates, and were transferred to 15 mL centrifuge tubes (VWR, Portugal), containing 2 mL of saline solution. To disaggregate the biofilms and resuspend the cells, the tubes were submitted to three alternate cycles of 30 s sonication (Ultrasonic Cleaner USC-T, 45 kHz, VWR International, Portugal), followed by 30 s of vortexing.

### Biofilm analysis

#### Optical coherence tomography (OCT)

Biofilms were imaged as described by Silva et al.^29^, directly from the 12-well plates with sterile saline solution, using spectral-domain Optical Coherence Tomography (OCT; Thorlabs Ganymede, Thorlabs GmbH, Germany) with a central wavelength of 930 nm^29^. The captured volume was 2.49 × 2.13 × 1.52 mm (y × z × x), consisting of 509 × 313 × 1024 pixels^3^. For each coupon, 2D and 3D imaging were performed with a minimum of five and three different fields of view (FoV), respectively. The acquired OCT images were processed with the software Biofilm Imaging and Structure Classification Automatic Processor (BISCAP)^31^, available at https://github.com/diogonarciso/BISCAP. In brief, for each 2D-OCT image, the pixels at the substratum were identified, and a threshold for the pixel intensity was calculated, enabling binarization of pixels as biomass or background, thereby distinguishing the biofilm from the liquid bulk phase^32^. The 2D image processing was extended to the 3D-OCT images, which correspond to 509 2D-OCT images as described by Narciso et al.^31^. BISCAP software was used to quantify the biofilm average thickness, compaction parameter and porosity. The specific definitions of the average thickness, compaction parameter and porosity can be found in Narciso et al.^31,32^. Briefly, the average thickness refers to the total length between the bottom and top of the biofilm. The compaction parameter, proposed by Narciso et al.^32^, measures the compactness of the biofilm; it represents the ratio between the continuous biomass pixels to the total number of pixels (biomass + water) between the bottom and top interfaces. The delivered values range from 0 to 1, where values closer to 1 correspond to very compact biofilms (with low empty spaces). The porosity was defined as the fraction of background voxels in the biofilm region, and varies between 0 and 1, as proposed by Narciso et al.^31^.

#### Peptide nucleic acid (PNA)—fluorescence in situ hybridization (FISH)

To track the spatial position of *L. pneumophila* inside biofilms, the PNA probe PLPNE620 (5′-CTG ACC GTC CCA GGT-3′) (Cambridge Research Biochemicals United Kingdom) was used, since it was successfully applied to detect the pathogen in past studies^28^. After rinsing with saline solution, coupons were allowed to air dry at room temperature. The PNA hybridization and washing step were performed according to Wilks et al.^28^. Control experiments were carried at each sampling timepoint to ensure that no cross-staining between *P. fluorescens* and *L. pneumophila* occurred, nor EPS staining. For that, control biofilms of *P. fluorescens* were hybridized with the PNA probe in the same conditions formerly described.

#### Episcopic differential interference contrast (EDIC)/epifluorescence (EF) microscopy

The stained coupons were examined using a Nikon Eclipse CFI60 episcopic differential interference contrast (EDIC) coupled with epifluorescence (EF) microscope, using a 50 × Plan APO objective (Best Scientific, UK). The EDIC channel was used to visualize the microscale structure of biofilms, while the TRITC channel was used to visualize and track the red labelled *L. pneumophila*. Representative images were taken over 20 fields of view and processed using ImagePro image capture software. The images were obtained with equal exposure times and gain values.

#### Confocal laser scanning microscopy (CLSM)

The stained coupons were also observed with a white light laser (WLL) at excitation wavelength of 565 nm and a 405-diode laser at excitation wavelength of 398 nm, using a 40 × glycerol objective lens in a Leica STELLARIS (Leica Stellaris, Leica Microsystems, Germany). A minimum of six stacks of horizontal plane images (512 × 512 pixels, corresponding to 387.5 × 387.5 µm) with a z-step of 0.36 µm were acquired for each sample. IMARIS 9.1 software (Bitplane, Switzerland) was used to create 3D projections of biofilm structures. The plugin COMSTAT2 from ImageJ was used to quantify the biovolume (µm^3^/µm^2^)^33^. The biovolume was defined as the overall volume of cells (µm^3^) divided by the substratum area, and it can be used to estimate how much biomass is in a biofilm^33^.

#### Quantification of sessile cells

To assess *P. fluorescens* culturability, serial dilutions were performed and plated in triplicate in plate count agar (PCA) (Oxoid, Portugal). Plates were incubated at 30 °C for 24 h for colony-forming units (CFU) enumeration. After assessing *P. fluorescens* culturability, biofilm suspensions were thermal treated (50 °C for 30 min) to eliminate *P. fluorescens* from the sample. The treated suspensions were spread onto the selective media BCYE-GVPC (buffered charcoal yeast extract supplemented with glycine, vancomycin, polymyxin and cycloheximide) agar and incubated at 37 °C up to 10 days to assess *Legionella* culturability.

#### *L. pneumophila* migration within the biofilm during the initial 24 h

The migration of *L. pneumophila* within the biofilm was followed over time during the first 24 h after spiking. Biofilm was sampled, labelled with the 16S rRNA PNA probe and imaged using CLSM, according to the previously described methods ("Peptide nucleic acid (PNA)—fluorescence in situ hybridization (FISH)" and "Confocal laser scanning microscopy (CLSM)" sections). The biofilms were analysed at 5 min, 15 min, 30 min, 2 h, 4 h, 6 h, 10 h, 20 h and 24 h after *Legionella* spiking.

### Statistical analysis

The experimental data were analysed using the software GraphPad Prism 9.0 for Windows (GraphPad Software, USA). Three independent experiments were performed. The mean and standard deviation (SD) for each set of results were calculated. Results were compared using an ANOVA single-factor statistical analysis and Student’s t-test. The level of significance was set for *p*-values < 0.05.

## Results

### *P. fluorescens *and *L. pneumophila* culturability

**Figure 1:**
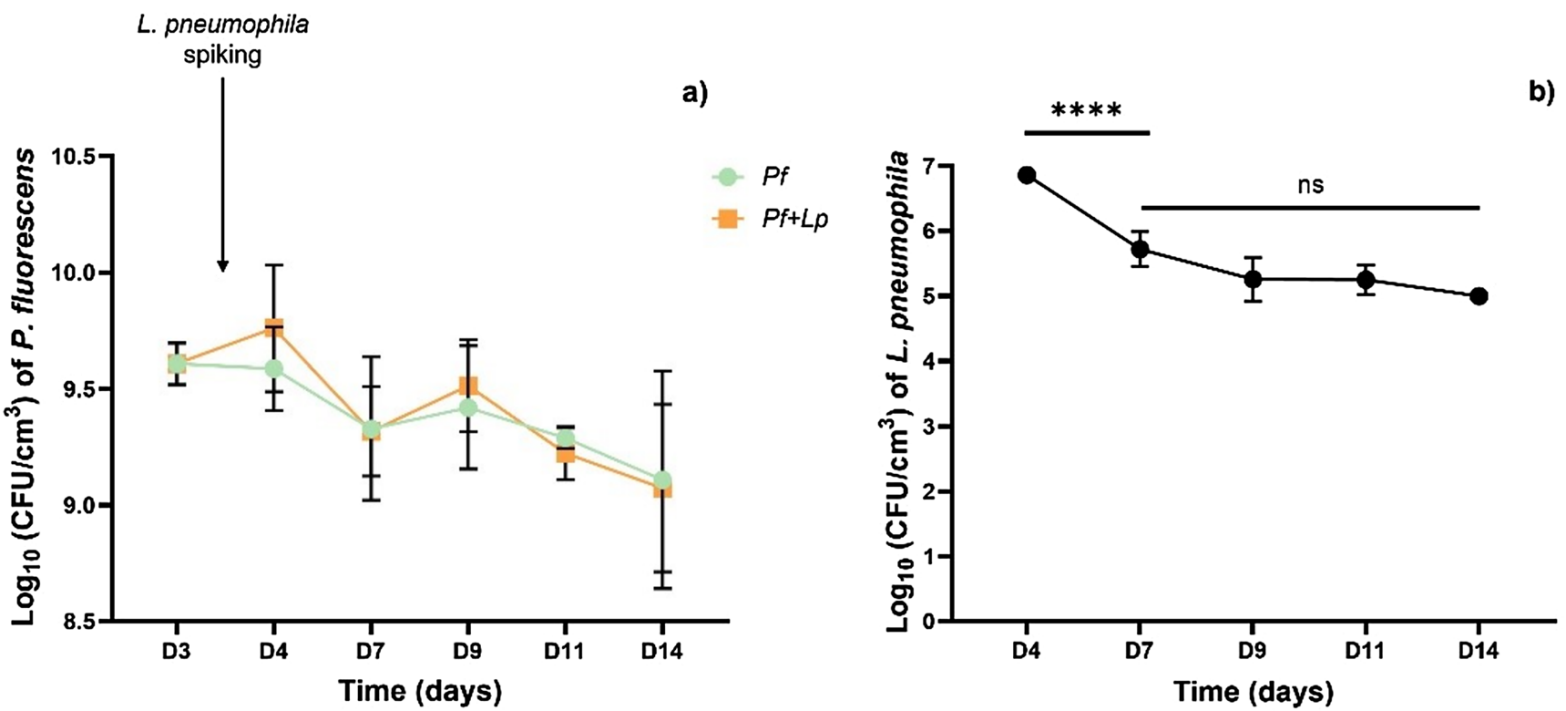
Bacteria culturability expressed per volume of biofilm (log_10_ CFU/cm^3^) (**a**) *P. fluorescens* and (**b**) *L. pneumophila* recovered from biofilm over time. The mean ± standard deviation is shown. Statistically significant differences are represented for *p* < 0.0001 by ****; ns: not statistically significant.

*P. fluorescens* culturability per volume of biofilm did not show statistically significant differences over time between the control biofilm (*P. fluorescens* alone—*Pf*) and those spiked at day 3 with *L. pneumophila* (*Pf* + *Lp*)—Fig. 1a. In both cases, the amount of *P. fluorescens* (~ 9 log_10_ CFU/cm^3^) did not significantly change between days 3 and 14 (*p* > 0.05). On the other hand, *L. pneumophila* was recovered for 11 days from the mixed biofilm of *Pseudomonas* and *Legionella*, but as shown in Fig. 1b, the culturable numbers of *L. pneumophila* per biofilm volume had 1-log reduction (*p* < 0.0001) between days 4 and 7 and maintained around 5 log_10_ CFU/cm^3^ until the end of each experiment. This reinforces the notion that *L. pneumophila* is able to colonize and persist (at least for 11 days) in *P. fluorescens* biofilms, confirming the previous work from Stewart et al.^13^.

### Biofilm mesoscale structure

**Figure 2:**
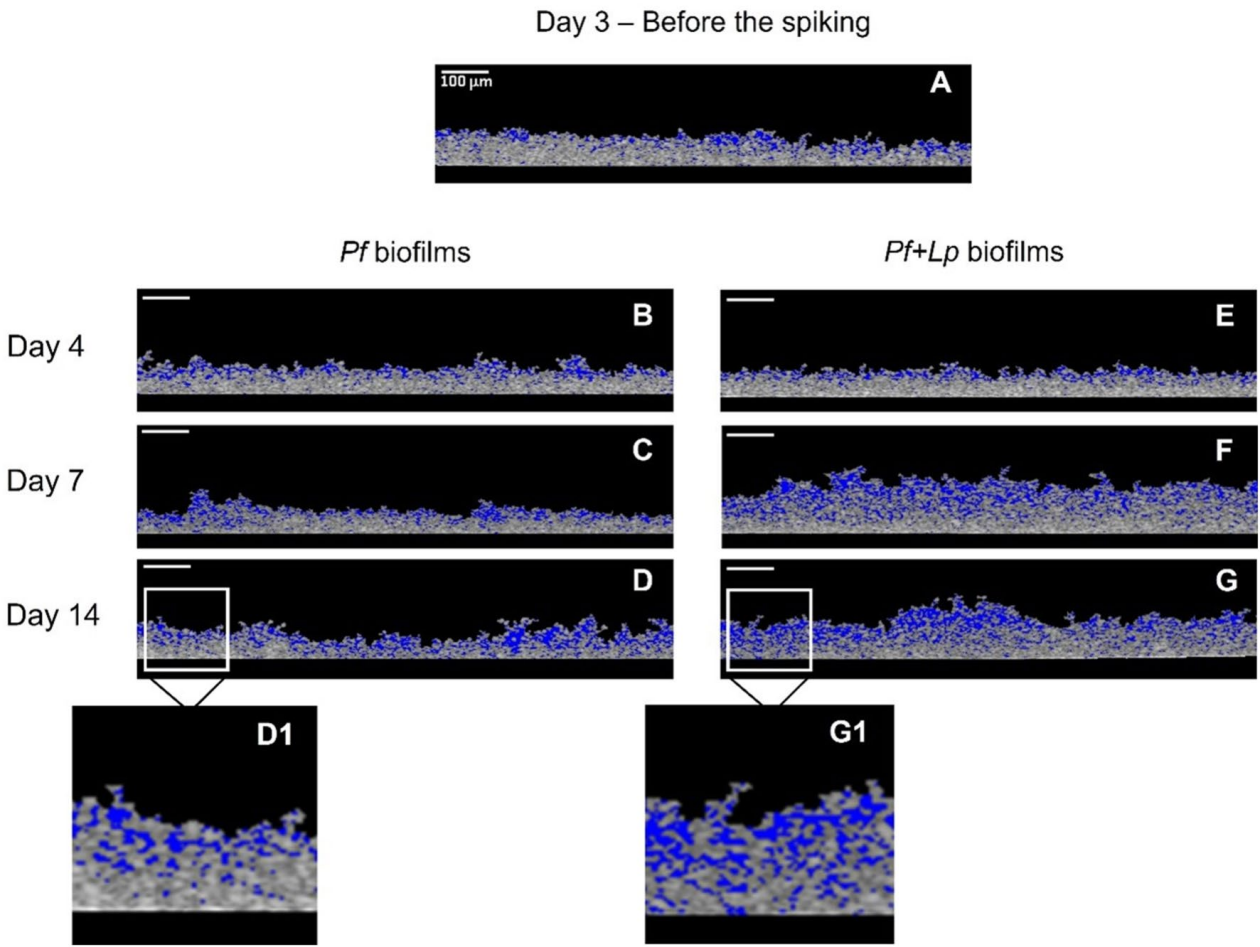
Representative images obtained by 2D-Optical Coherence Tomography (OCT) of 3-, 4-, 7- and 14-day biofilms not spiked (left side) and spiked (right side) with *L. pneumophila*. White scale bars are 100 µm. Specific areas of Fig. 2D (D1) and 2G (G1), marked with a white rectangle, were × 12 enlarged. The empty spaces within the biofilm structure are colored in blue.

The mesoscale structures of the control biofilms of *P. fluorescens* (*Pf*—without *L. pneumophila*) were compared with those spiked with *L. pneumophila* (*Pf* + *Lp*) on day 3. Figure 2 depicts representative 2D-OCT biofilm images for both conditions (*Pf* and *Pf* + *Lp* biofilms).

When analyzing the control *P. fluorescens* biofilm mesoscale structure over time, it can be seen that the regular and flat structure observed on day 3 (Fig. 2A) is similar to the one found on day 4 (Fig. 2B). Over time, *P. fluorescens* control biofilms (Fig. 2C and D) tend to become more irregular and exhibit more empty spaces (colored in blue). A similar behavior is observed for the *P. fluorescens* biofilms spiked with *L. pneumophila*, except that, for longer incubation periods, the spiked biofilms (Fig. 2F and G) tend to be significantly thicker than the control biofilms, and show increased empty channels. Not surprisingly, the area occupied by the empty channels is more pronounced in the top of the biofilm than in the bottom, for the control and spiked biofilms.

**Figure 3:**
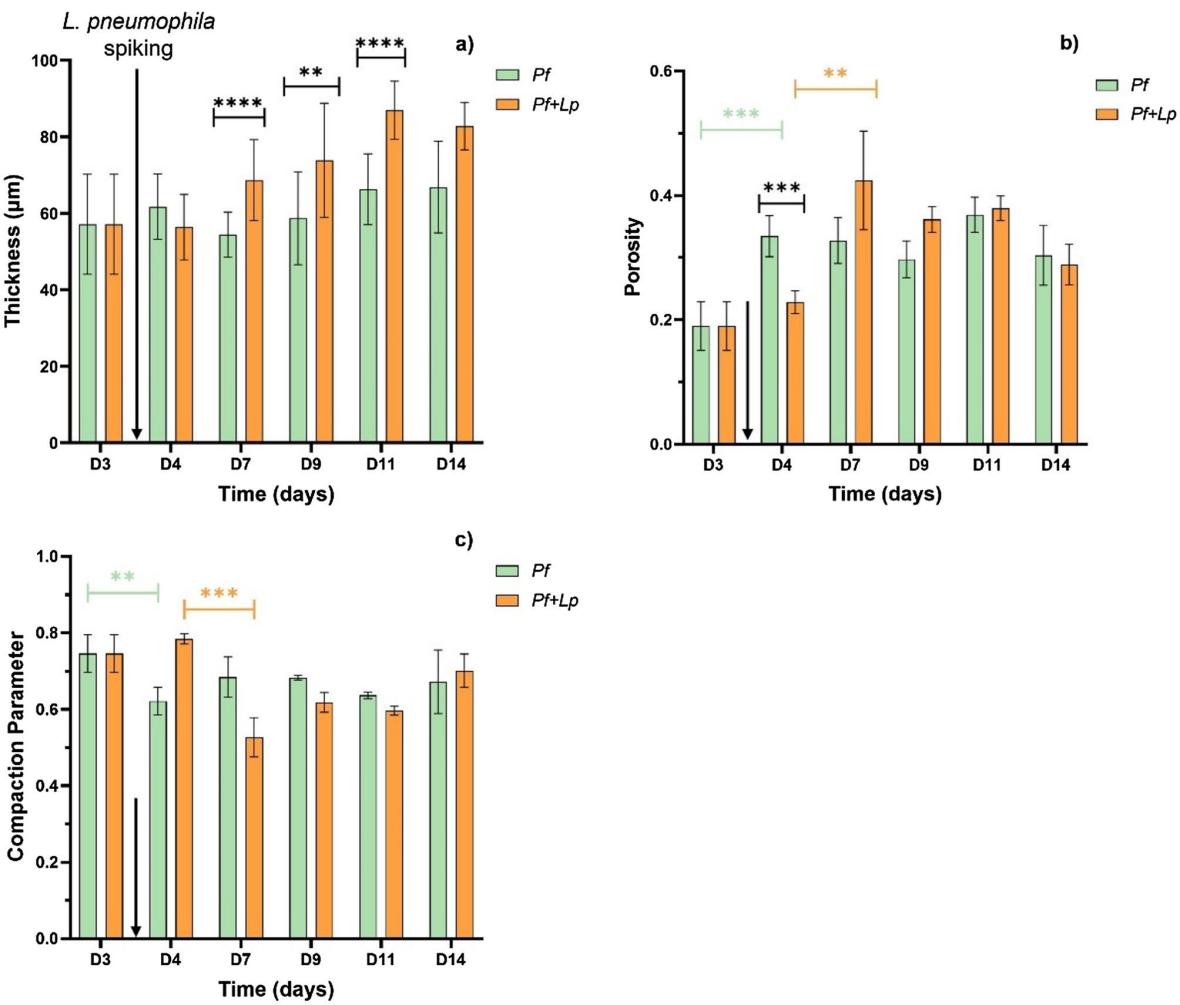
Thickness (**a**), porosity (**b**) and compaction parameter (**c**) of the control (*Pf*) – green bars and spiked (*Pf* + *Lp*) – orange bars biofilms over 14 days. Values were extracted from 3D-OCT images with the BISCAP software. The mean ± standard deviation is shown. Statistically significant differences are represented for *p* < 0.05 by *, < 0.01 by **, < 0.0005 by *** and < 0.0001 by ****. Error bars in black, green and orange refer to significant differences between control and spiked biofilms, between the control biofilms and between the spiked biofilms, respectively. *L. pneumophila* spiking is indicated by an arrow.

Based on the 3D-OCT biofilm images and using the BISCAP software^31^ the following biofilm structural parameters were quantified: thickness (Fig. 3a), porosity (Fig. 3b) and compaction parameter (Fig. 3c).

No significant changes were observed in the thickness profile of the *P. fluorescens* control biofilms (Fig. 3a, green bars) which was found to be 61 ± 11 µm over the 14 days experimental period. The other mesoscale parameters showed significant changes from days 3 to 4 (*p* < 0.05): while porosity (Fig. 3b) increased, the compactness of the biofilm has been reduced (Fig. 3c). From day 4 until the end of the experiment, the above mentioned parameters remained stable, suggesting the biofilm structure reached the plateau^34^.

Upon *L. pneumophila* spiking to the *P. fluorescens* biofilms (*Pf* + *Lp*), no significant changes in thickness were noticeable between days 3 and 4, as shown in Fig. 3a (orange bars). However, from days 7 to 14, biofilms with *L. pneumophila* became significantly thicker than the ones of *P. fluorescens* alone (*p* < 0.0001), reaching the highest thickness of 90 µm by day 11. The porosity and compactness did not change (*p* > 0.05) between days 3 and 4 (*Pf* + *Lp*, orange bars). Changes were only noticeable later, by day 7, as the porosity increased (*p* < 0.05) and the compaction decreased (*p* < 0.05), to values like the ones from the non-spiked biofilms (*Pf*).

### *Legionella* spatial location

**Figure 4:**
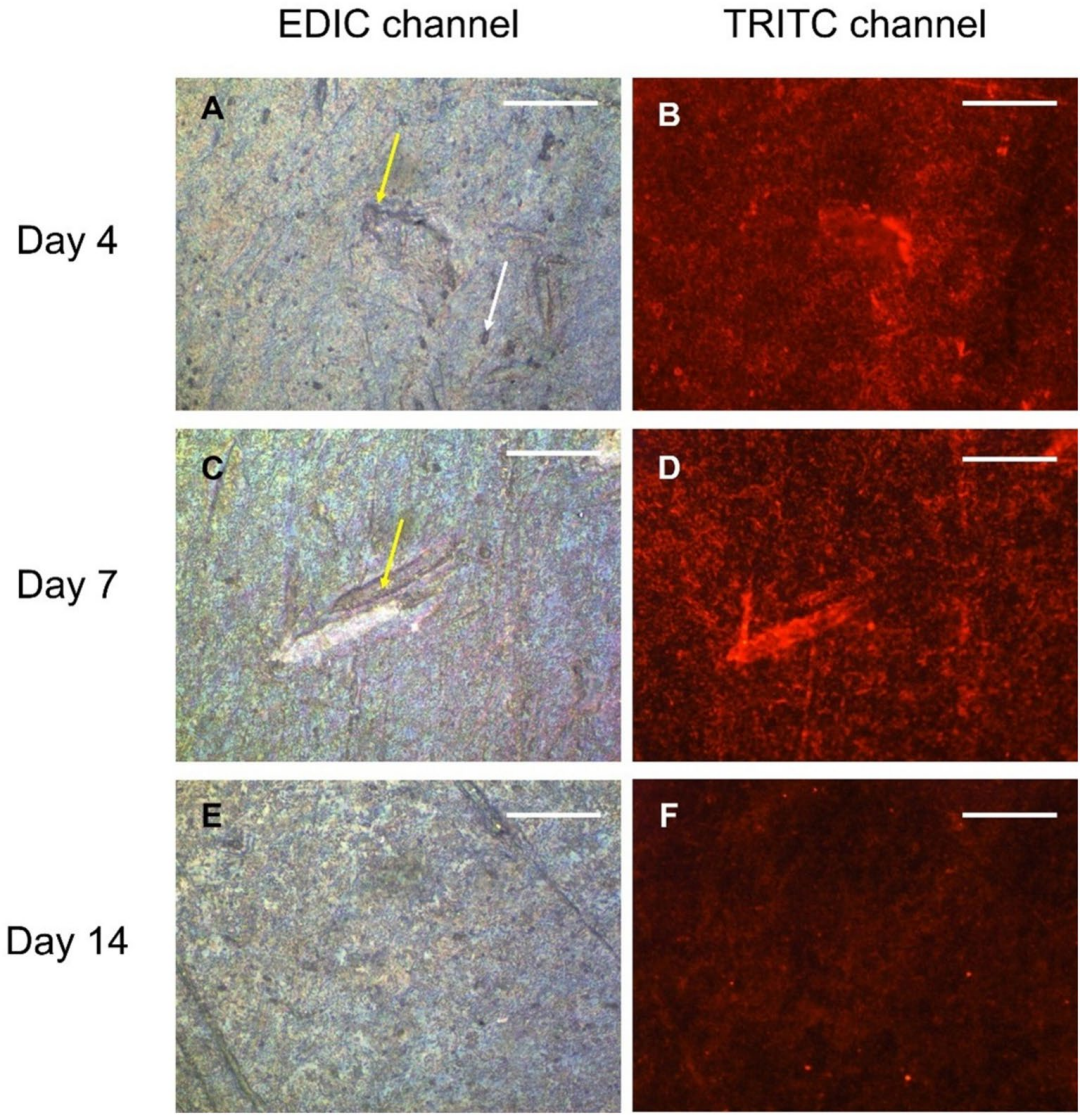
Representative EDIC/EF images of 4-, 7- and 14-days biofilms spiked with *L. pneumophila*; the latter were stained with a PNA probe (in red). Biofilms were visualized using the EDIC channel (images **A**, **C** and **E**) and using a TRITC filter for fluorescence (images **B**, **D**, and **F**). White arrows indicate microcolonies and yellow arrows indicate areas highly colonized. Bars represent 10 µm. Magnification × 500. A representative image of the control of *P. fluorescens* biofilm stained with the PNA probe is provided in Supplementary Information (Fig. S1).

**Figure 5:**
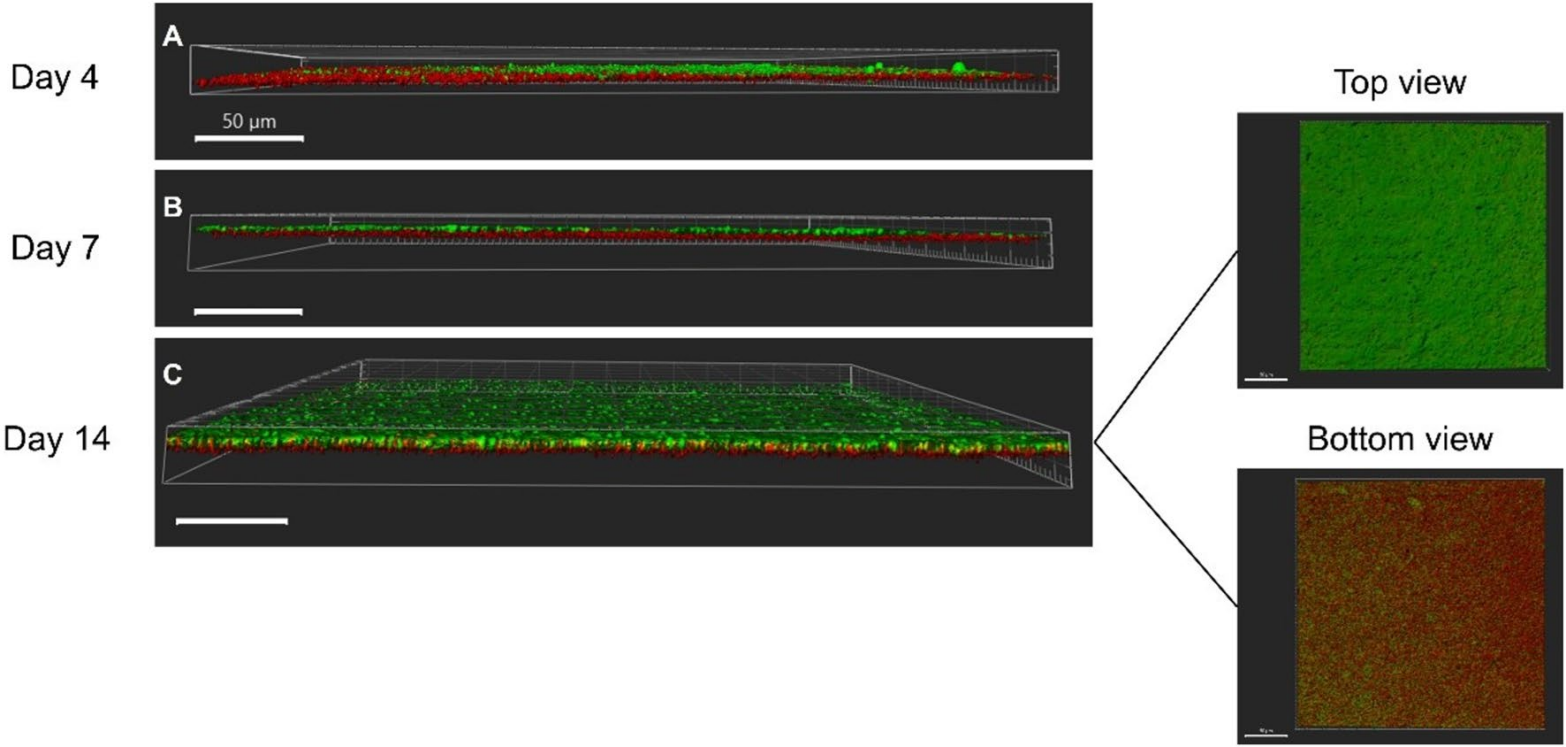
Representative CLSM images of 4-, 7- and 14-days biofilms spiked with *L. pneumophila*; The latter was stained with a PNA probe (in red). The confocal images are 3D projections obtained using IMARIS, and the white scale bars are 50 µm. A representative image of the control *P. fluorescens* biofilm stained with the PNA probe is provided in Supplementary Information (Fig. S2).

To study the spatial location of *L. pneumophila* within the *P. fluorescens* biofilms, the microscale structure of the spiked biofilms was characterized by episcopic differential interference contrast microscopy (EDIC) with epi-fluorescence (EF) and by Confocal Laser Scanning Microscopy (CLSM). *L. pneumophila* is labelled red through the specific 16S rRNA PNA probe (PLPNE620). Representative images of *P. fluorescens* biofilms stained with the same PNA probe and visualized at the EDIC/EF (Fig. S1) and CLSM (Fig. S2) are provided in the Supplementary Information. These images show that there is no cross-staining between the bacteria nor any interaction with the biofilm EPS (no red signal is observed). Figures 4 and 5 show representative EDIC/EF and CLSM images of the spiked biofilms, respectively, and show that *L. pneumophila* was widespread within the coupons, and also emphasize the success of bacteria in colonizing the *P. fluorescens* biofilm.

The EDIC/EF microscopy images allowed to qualitatively characterize the biofilm microscale structure and to visualize the predominant location of *L. pneumophila* within it. Direct observation of biofilms 24 h after the *L. pneumophila* (day 4) spiking, EDIC/EF imaging revealed the presence of microcolonies (Fig. 4—white arrows) and the diffuse fluorescence surrounding them is indicative of eDNA in the accumulating EPS. The presence of microcolonies was further confirmed with the OCT since each of the individual black dots are too large to be individual bacteria and more likely to be microcolonies (approximately 10–20 microns in diameter). In general, from days 4 to 14, there were some highly colonized areas (Fig. 4—yellow arrows) separated by others with less biofilm density, showing the heterogeneous nature of biofilms. Biofilms showed increased thickness with time, which is particularly noticeable by day 14 (Fig. 4E). In this figure, biofilm microcolonies seem to be brighter and more well-defined than in previous days, which reflects the growth of the microcolonies and the expected higher rRNA content present in the biofilm.

*L. pneumophila* red fluorescing cells can also be seen (under the TRITC filter), evidencing its widespread distribution within the biofilm. Regions, where the coupon was scratched or with some more prominent biofilm aggregates, had massive *L. pneumophila* clumps. Some water channels were also observed in the biofilm, but no significant amounts of *L. pneumophila* were observed near such water channels. The intensity of the red fluorescing cells (Fig. 4B, D and F) seems to become faint over time (particularly by day 14).

The detailed investigation of the *L. pneumophila* spatial position within the *P. fluorescens* biofilm was established via confocal imaging. The three–dimensional reconstructions of the biofilms—Fig. 5—revealed the presence of *P. fluorescens* (observed as green due to the autofluorescence conferred by self-produced pigments^35,36^) and *L. pneumophila* in very similar proportions. Furthermore, *L. pneumophila* was mostly located in the bottom layers of the biofilm. This was observed for the whole experimental period.

### Quantification of the biofilm microscale structure

**Figure 6:**
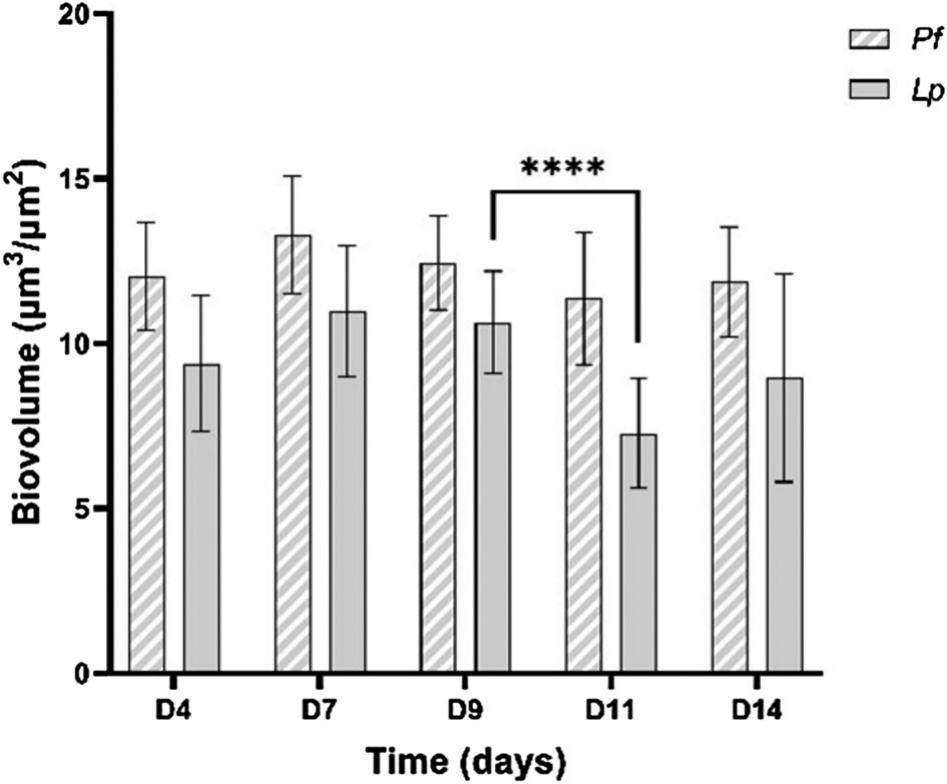
Biovolume of *P. fluorescens* and *L. pneumophila* in spiked biofilms (*Pf* + *Lp*) developed under 14 days. Values were extracted from confocal images with the COMSTAT plugin. The means ± standard deviations are shown. Statistically significant differences are represented for *p* < 0.0001 by ****.

The biovolume of *P. fluorescens* and *L. pneumophila* in the spiked biofilms (Fig. 6) were determined by CLSM from days 4 to 14.

The biovolume of *P. fluorescens* in the mixed *Pf* + *Lp* biofilms remained constant (12 ± 2 µm^3^/µm^2^) from days 4 to 14 (*p* > 0.05), while the biovolume of *L. pneumophila* increased (not statistically significant) until day 9 (11 ± 1 µm^3^/µm^2^) and became significantly lower (*p* < 0.05) at day 11 (7 ± 2 µm^3^/µm^2^).

### *L. pneumophila* migration within the biofilm during the initial 24 h

**Figure 7:**
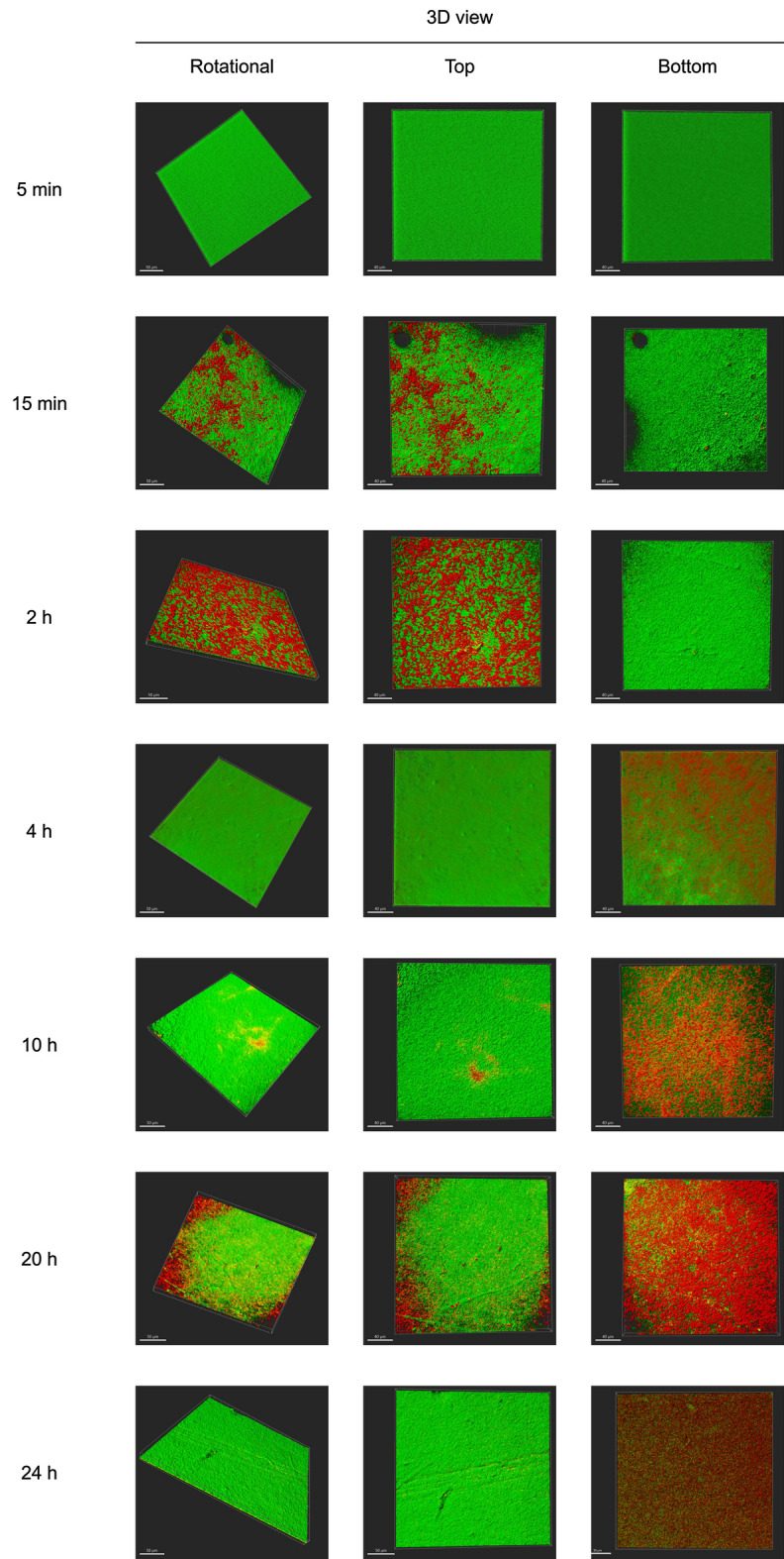
Representative CLSM images of biofilms 5 min, 15 min, 2 h, 4 h, 10 h, 20 h and 24 h after *L. pneumophila* spiking; The confocal images are 3D projections obtained using IMARIS, and the white scale bars are 50 µm.

The migration of *L. pneumophila* within the *P. fluorescens* biofilm was monitored over a 24 h period after *L. pneumophila* spiking, using confocal imaging (Fig. 7). No *L. pneumophila* was observed in the 5 initial minutes after the spiking. A thin layer of *L. pneumophila* was detected on the top surface of the biofilm 15 min after spiking. Over time, an increase in *L. pneumophila* on the top of the biofilm was observed, suggesting an accumulation of the bacteria. By the 4 h mark, a significant amount of *L. pneumophila* started to appear in the bottom layers of the biofilm, simultaneously with a bacterial decrease on the top. This migration continued progressively, with *L. pneumophila* becoming predominantly located at the bottom of the biofilm by the end of the 24 h observation period.

## Discussion

*L. pneumophila* entrance in the 3-days *P. fluorescens* biofilm was evaluated regarding the impact on the biofilm structure and on the bacteria positioning over 11 days.

### *L. pneumophila* colonization of the *P. fluorescens* biofilm—impact on the biofilm structure

When *L. pneumophila* colonizes the *P. fluorescens* biofilms, they maintained their mesoscale structure (quantified through thickness, porosity, and compaction parameter of the 3D-OCT images) as no significant differences were found between days 3 and 4 (before and 24 h after *L. pneumophila* spiking, respectively)—Fig. 3 (orange bars). Differences in the *Legionella* spiked biofilms structure were only noticeable later (when sampling the biofilm at day 7), as they tended to rearrange into similar characteristics as those from the control (*P. fluorescens* alone) biofilms. Lee et al.^37^ reported a delay in biofilm development, concluding that the development of mixed-species is slower (1- or 2-day delay) than single-species biofilms. The control biofilm (*P. fluorescens* only) rearranged structurally between days 3 and 4 (Fig. 3, green bars), and then remained stable, suggesting that the biofilm development reached its plateau by day 4.

However, thickness followed a different trend: from days 7 to 14, the spiked biofilms became progressively thicker (~ 30%) than the *Pf* controls (Fig. 3a, green bars). A similar behaviour was found by Koh et al.^38^ who described that the thickness of *P. aeruginosa* biofilms exposed to a waterborne pathogen, *Cryptosporidium parvum*, increased when compared to the control biofilms. Also, Puga and colleagues^39^ reported that spiking *Listeria monocytogenes* to pre-established *P. fluorescens* biofilms led to an EPS matrix over-production. According to other authors, mixed-species biofilms might have an increased biomass production^37,40^, which can be related to events of space optimization due to different bacterial interactions^41^.

The other mesoscale characteristics of the biofilms (including porosity and compaction parameter) suggest that regardless of the *Legionella* presence, the dominant biofilm structure is the one from the *P. fluorescens—*the first colonizer. In addition, the present results show that the cell density of *P. fluorescens* (Fig. 1a) was not significantly affected by the presence of *L. pneumophila*. Pang et al.^42^ while studying the colonization of *P. fluorescens* biofilms by *L. monocytogenes* also concluded that *P. fluorescens* cell density did not change with the presence of *L. monocytogenes*.

The observed dominance of *P. fluorescens* over *L. pneumophila* in the biofilm may be related with the fact that *P. fluorescens* is a well-known EPS producer strain^20,42,43^. It has been previously reported that microorganism producers of EPS have competitive advantages over other bacteria if they are the first colonizers^44^. Some authors argue that *Legionella* is able to form biofilms on its own under very well-defined laboratory conditions^45,46^, but with no significant amounts of EPS^46^. However, under real environmental scenarios, *Legionella* colonizes pre-established biofilms, as a secondary colonizer^5^. Furthermore, the large amounts of EPS produced by *P. fluorescens* might enhance the physical fixation/entrapment of *L. pneumophila* and will allow the establishment of more robust biofilms with increased cohesion^39,47^, arguably more difficult to suffer slough-off.

### *L. pneumophila *location within the *P. fluorescens* biofilm

Results showed that *L. pneumophila* successfully colonized and persisted in a *P. fluorescens* biofilm at least for 11 days.

The EDIC images showed that PNA-*L. pneumophila* signal became faint over time, which seems to be consistent with the *Legionella* biovolume (Fig. 6) and culturability (Fig. 1b) decrease over time. In all situations this might be a consequence of *L. pneumophila* entering a non-culturable but viable state (VBNC). It is reported that VBNC cells have lower metabolic activity and lower levels of rRNA^48,49^. If the amount of rRNA decreases, and since the PNA probe binds specifically to 16S rRNA molecules, one might expect that the intensity of the signal (observed as a red color) will also decrease^48,50^. Former studies showed that there are a vast number of 16S rRNA molecules per bacterium compared to copies of the gene^51,52^. Thus, the bright and further decrease in the PNA-FISH signal is arguably due to decreasing 16S rRNA content and not from the very low number of copies of the 16S rRNA chromosomal gene. The ability of *Legionella* to enter into the VBNC state has been demonstrated by several authors^53–55^. Gião et al.^56^ and Alleron et al.^57^ induced *L. pneumophila* cells into VBNC state through chlorine and monochloramine exposure, respectively. Indeed, the former remained infective in an *Acanthamoeba* animal model. Other studies also concluded that under a low nutrient environment, *Legionella* would lose its culturability^58^, and that VBNC cells exhibit smaller cell sizes^59,60^. An alternative explanation for the faint signal might be that, over time*, L. pneumophila* is washed-off of the biofilm, as the medium is replaced every 2 days.

Regarding the spatial positioning of the bacteria, the CLSM images (Fig. 5) show that bacteria were essentially positioned in two distinct layers. While *L. pneumophila* was positioned in the bottom of the biofilm, *P. fluorescens* was located in the upper layers (Fig. 5). Two distinctive physiological aspects between both bacteria are related to the oxygen consumption and nutrients uptake. While *P. fluorescens* metabolizes carbon sources and is aerophilic^61^, *L. pneumophila* has very specific nutritional requirements and behaves as a microaerophilic microorganism^62^, thus growing in the presence of oxygen but better at lower oxygen levels. Since the transport of nutrients and oxygen is higher at the biofilm top interface^63^, the relative positioning of *Pseudomonas* and *Legionella* inside the biofilm is a win–win situation for both bacterial species. This also explains why *L. pneumophila* is not placed around water channels (observed in the EDIC/EF imaging—Fig. 4), as the primarily function of water channels is to favor mass transport (nutrients, oxygen, waste-products, etc.) between the biofilm and the surrounding liquid^64^. And expectedly higher oxygen and nutrients concentrations might be found on those areas^11^. It is not surprising though that *Legionella* is located at the bottom layers of the biofilm where micro-environments with lower oxygen levels can be found. Additionally, it has been demonstrated that the EPS producer cells and their descendants (in the case of the present study—*P. fluorescens*) will be positioned in the biofilm top layers, keeping privileged access to nutrients and oxygen and allowing such bacteria to dominate the biofilm^65^. Indeed, the OCT imaging (Fig. 2) demonstrated that most of the empty spaces—that are linked to events of mass transfer—are located in the upper layers of the biofilm^66^. This also supports the former conclusions of the present work that by the middle of the experimental biofilms colonized by *L. pneumophila* presents the same mesoscale structure properties (except for thickness) as the one from the *P. fluorescens* control biofilm.

Finally, from the *Legionella* perspective, being at the bottom of the biofilm (the EDIC/EF imaging showed that many cells were in the scratches of substratum material), *Legionella* will be more protected than in the top layers against external harshness like biocides or thermal shocks. There are several studies demonstrating the ability of *P. fluorescens* biofilms to shield pathogens^39,67,68^.

### How long does *L. pneumophila* need to reach the bottom of the *P. fluorescens* biofilm?

The time-lapse representative CLSM images of *L. pneumophila* colonization of the pre-established *P. fluorescens* biofilm over the initial 24 h after *L. pneumophila* spiking (Fig. 7) show that *L. pneumophila* starts to adhere, to a greater extent, to the top of the biofilm within 15 min after spiking. It is somehow surprising that no *L. pneumophila* was observed in the first 5 min, as the experiment was conducted under stagnation (no flow) conditions. Former work demonstrated that sedimentation significantly affects bacterial attachment and mass transfer, even under low flow conditions^69,70^. Under no-flow conditions, the sedimentation effect is even higher, and the entire biofilm was surrounded by *Legionella*. Therefore, the fact that *L. pneumophila* took between 5 and 15 min to adhere to the top layer of the *P. fluorescens* (Fig. 7, Top, 15 min), is likely due to the multiple adaptation strategies that *Legionella* can undergo. Several studies show that the morphological changes of *Legionella* appendages are critical to the interactions within host-protozoa and allow the bacteria to switch between the replicative and transmissive phases^71^. The study from Abdel-Nour et al.^72^ also shows that adhesins, in particular, collagen-like adhesin is important for *Legionella* attachment to surface, biofilm formation and auto-aggregation.

Once *L. pneumophila* interacts with the top layer of the biofilm it quickly (between 2 and 4 h) reaches the bottom of the *P. fluorescens* biofilm. Considering that the pre-established 3 days biofilm have an average thickness of ~ 58 µm, the average linear migration speed of *L. pneumophila* across the biofilm is ~ 22 µm/ h. This migration speed is consistent with the range proposed by Picioreanu et al.^73^ for the computational model simulation of *P. aeruginosa* biofilm formation, which accounted with many factors, including cells motility and twitching motility. Albeit it is important to remark that in the present study, *L. pneumophila* was not the first colonizer and already encountered a pre-established thick biofilm, with high cellular density (~9 log_10_ CFU/cm^3^) and a very well organized mesoscale structure (Fig. 2A), which could have been a constraint to *L. pneumophila* and migration. Puga et al.^39^ attributed the differences between the colonization of 48 h pre-established *P. fluorescens* biofilms by *L. monocytogenes* formed under different conditions to the physical impediment bacteria face when entering different structures of the already established biofilms. It seems that apart from the hypotheses already discussed regarding the distinctive physiological aspects between the two bacteria species (nutrient and oxygen requirements), *L. pneumophila* might had also taken advantage of the empty spaces found in the *P. fluorescens* biofilm (Fig. 2A—colored in blue) to quickly move across the biofilm and reach its bottom. As previously discussed, no significant changes were observed at the mesoscale structure of the biofilm (Figs. 2 and 3) reinforcing the idea that *L. pneumophila* took advantage of the already existing biofilm structure rather than creating transient biofilm structures (like pores or channels) as reported in other works^74^.

After 4 h, the significant decrease of *L. pneumophila* in the top layer of the biofilm is arguably related to sedimentation and with the fact that *L. pneumophila* keeps moving across the biofilm since a significant increase of red stained *L. pneumophila* cells is observed in the bottom of the biofilm. Between 20 and 24 h all the *L. pneumophila* is positioned in the bottom layer of the *P. fluorescens* biofilm (Fig. 7), in a very high concentration (~7 log_10_ CFU/cm^3^, Fig. 1b). The 24 h *L. pneumophila* concentration in the biofilm and in the bulk (~8 log_10_ CFU/mL), raises the question of whether *L. pneumophila* is or not able to replicate within a mono-specie biofilm even if it is over a small timespan. A proper answer to this question requires further investigation. Of note is that the *L. pneumophila* numbers provided were obtained by culturability, thus likely reflecting an underestimation the true amounts of bacteria in the system.

The present work brings new insights for the discussion about *Legionella* and biofilms interactions concerning the structural changes and relative location of *L. pneumophila* within the *P. fluorescens* biofilm. Although the experimental design does not aim to mimic the interactions of biofilm-*Legionella* in engineered water systems, it provides an expedite approach to tackle some fundamental questions regarding such interactions. The combination of micro and mesoscale techniques provided significant and complementary information that can be used in future works and in real studies. In this scope, it worth to highlight that OCT imaging showed to be a powerful non-staining technique that rapidly describes the biofilm 3D meso-scale structure, microcolonies accumulation and water filled areas.

It is important to remark that the results obtained in the present study might be different concerning the pre-established biofilm species used or the *Legionella* species/strains considered or the introduction of host cells.

Finally, the proposed experimental model offers to the scientific community a platform to study, in a systematic way, several questions related to mechanistic and physiological aspects of *Legionella*-biofilms interactions, including virulence, transmission, the behaviour of mutants (among many others) which might allow, in the future, to better understand the bacteria dynamics in the complexity and variability of real systems.

Future work is focused on answering to some of the questions raised during this study regarding whether *L. pneumophila* replicates or not in the biofilm and whether it enters VBNC states or wash-off from the biofilm over time. Since biofilm detachment is critical from a public health perspective of legionellosis prevention the model will also be revised to consider this aspect in future works.

## Conclusions

Biofilms are a key ecological niche for *Legionella* persistence in water systems, although the microbial interactions between them are still poorly understood. The laboratory model developed in this study deciphered some of the interactions of *L. pneumophila* and *P. fluorescens* biofilms. The main findings of this work are: (a) the overall dominant biofilm structure is the one provided by *P. fluorescens*, regardless of the *L. pneumophila* colonization; (b) the spiked biofilms are thicker than the ones from *P. fluorescens* alone; (c) *L. pneumophila* reaches in 2–4 h the bottom of the biofilm, were it is preferentially positioned over the 11 days of the trial, thus being more protected from external stressors, and (d) both PNA-labelling and *L. pneumophila* culturability suggest that by the end of the experiment *Legionella* might be entering a VBNC state for stress survival.

### Supplementary Information

Supplementary Figures.

## Data Availability

The datasets that support the findings of this study are available from the corresponding author on reasonable request.
