# Supplementary material for: Legionella colonization and 3D spatial location within a Pseudomonas biofilm
Source: Sci Rep. 2024 Jul 22;14:16781. doi: 10.1038/s41598-024-67712-4 (PMC11263398; doi:10.1038/s41598-024-67712-4)
Supplement: Supplementary file 1 — Supplementary Figures. [file 41598_2024_67712_MOESM1_ESM.docx]

***Legionella* colonization and 3D spatial location within a *Pseudomonas* biofilm**

Ana Rosa Silva^1,2^, Luis F. Melo^1,2^, C. William Keevil^3^, Ana Pereira^1,2*^

^1^LEPABE - Laboratory for Process Engineering, Environment, Biotechnology and Energy, Faculty of Engineering, University of Porto, Rua Dr. Roberto Frias, 4200-465 Porto, Portugal

^2^ALiCE - Associate Laboratory in Chemical Engineering, Faculty of Engineering, University of Porto, Rua Dr. Roberto Frias, 4200-465 Porto, Portugal

^3^School of Biological Sciences, University of Southampton, Southampton, United Kingdom

**Supplementary Material**

**
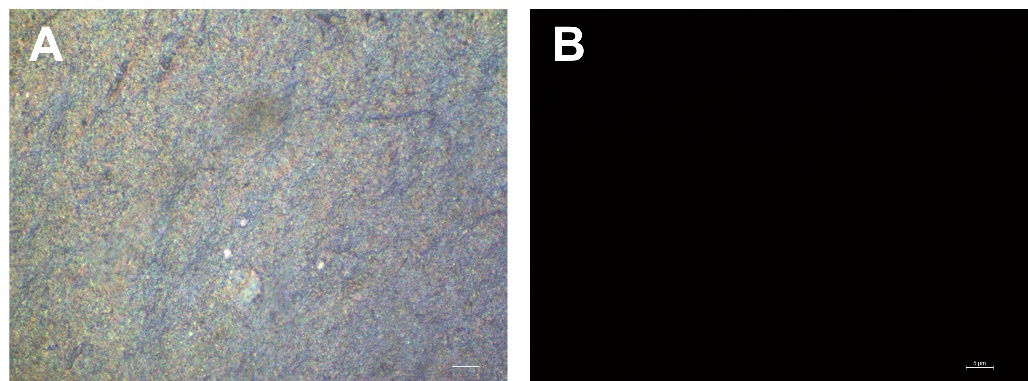
**

**Figure S1.** Representative EDIC/EF image of 14-days *P. fluorescens* biofilms (control – **without** *L. pneumophila*); the control biofilms were hybridized with the PNA probe PLPNE620 (red stain; specific for *L. pneumophila*). Biofilms were visualized using the EDIC channel (image A) and using a TRITC filter for fluorescence (image B). Bars represent 5 µm. Magnification ×1000.


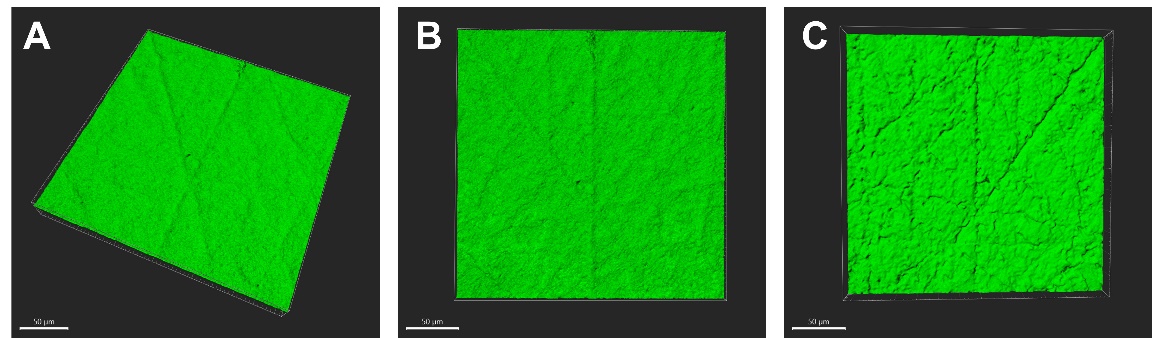


**Figure S2.** Representative CLSM image of 14-day *P. fluorescens* biofilms (control – **without** *L. pneumophila*); the control biofilms were hybridized with the PNA probe PLPNE620 (red stain; specific for *L. pneumophila*). The confocal images are 3D projections obtained using IMARIS (A – rotational view, B – top view and C – bottom view), and the white scale bars are 50 µm.
